# Chromosome microarray analysis in the investigation of children with congenital heart disease

**DOI:** 10.1186/s12887-017-0863-3

**Published:** 2017-05-04

**Authors:** Xiao-li Wu, Ru Li, Fang Fu, Min Pan, Jin Han, Xin Yang, Yong-ling Zhang, Fa-tao Li, Can Liao

**Affiliations:** Prenatal Diagnostic Center, Guangzhou Women and Children’s Medical Center, Guangzhou Medical University, No. 9 Jinsui Road, Guangdong, China

**Keywords:** 15q11.2 deletion, 1q43-q44 deletion, Chromosome microarray analysis, Congenital heart disease, Copy number variation, Microdeletion/microduplication

## Abstract

**Background:**

Our study was aimed to explore the clinical implication of chromosome microarray analysis (CMA) in genetically etiological diagnosis of children with congenital heart disease (CHD).

**Methods:**

A total of 104 children with CHD with or without multiple congenital anomalies (MCA) or intellectual disabilities/developmental delay (ID/DD) but normal karyotype were investigated using Affymetrix CytoScan HD array.

**Result:**

Pathogenic copy number variations (PCNVs) were identified in 29 children (27.9%). The detection rates in children with simple CHD and complex CHD were 31.1% (19/61) and 23.2% (10/43), respectively. The detection rates of PCNVs were 17.9% (7/39), 20% (5/25), 63.2% (12/19) and 23.8% (5/21) in isolated CHD, CHD plus MCA, CHD plus ID/DD, CHD plus MCA and ID/DD, respectively. The PCNVs rate of CHD plus ID/DD was significantly higher than that of isolated CHD. Two genomic loci including 15q11.2 deletion and 1q43-q44 deletion were considered as CHD locus. The *DVL1, SKI, STIM1, CTNNA3* and *PLN* were identified as candidate genes associated with CHD phenotypes.

**Conclusion:**

CMA can increase the diagnostic rate and improve the etiological diagnosis in children with CHD. We suggest CMA as a first-tier test in children with CHD, especially in children with CHD plus ID/DD.

## Background

Congenital heart disease (CHD) is one of the most common birth defects. The incidence of CHD in the neonate is 8-9/1000, and nearly 1.35 million CHD neonates were born every year in the whole world [1]. Despite improvement of various treatment measures, CHD is still one of the major causes of children mortality.

The causes of CHD include non-genetic factors and genetic factors. Non-genetic factors include: environmental factors, maternal exposure and infection. Chromosomal causes of CHD include chromosome aneuploidies, like trisomy 21, and copy number variations (CNVs). Chromosomal aneuploidies represent 12.5% of CHD causes [2]. The spectrum of CHD-related CNVs ranges from recurrent microdeletion and microduplication syndromes, like DiGeorge syndrome (22q11 deletion syndrome) and Williams-Beuren syndrome (7q11.23 deletion syndrome), which are associated with a distinct clinical recognizable phenotype, to rare CNVs, flanked by unique breakpoints [3–5].

The resolution of conventional karyotype analysis is limited to 5 Mb or larger genomic imbalances [6]. The drawback of fluorescence in situ hybridization (FISH) lies in its a targeted approach to detect chromosomal defects, rather than a genome-wide screening method like microarrays or MLPA [7]. Chromosome Microarray Analysis (CMA) is a routine technique in clinical molecular testing nowadays, which contains two types of arrays: oligonucleotide arrays and Single Nucleotide Polymorphism arrays (SNP arrays). Both the arrays could detect genome-wide CNVs. Moreover, SNP arrays can detect the mosaicism, triploid, loss of heterozygosity (LOH) and uniparental disomy. In 2010, the American College of Medical Genetics issued practice guidelines for CMA, and pointed out that CMA was recommended as a first-tier test for postnatal patients with multiple congenital anomalies (MCA), intellectual disabilities/developmental delay (ID/DD) and autism spectrum disorders [8]. Recently, CMA has been successfully applied to detect CNVs in patients with CHD, which confirmed the relationship between chromosome microdeletion/microduplication and CHD [9–19].

In this study, we present the results of genome-wide high resolution SNP array analysis in 106 children with CHD in the Chinese cohort, to explore the clinical implication of CMA in genetically etiological diagnosis of CHD.

## Methods

### Subjects and sample

In this study, we collected children diagnosed with CHD with or without MCA or ID/DD and normal karyotype at Guangzhou Women and Children’s Medical Center from January 2012 to December 2014. All the children were investigated by the complete cardiac evaluation, echocardiogram, medical history, physical examination, and/or operative reports. Considering patent ductus arteriosus (PDA) was common in children with CHD, the child with PDA was selected only if he/she was born at >37 weeks’ gestational age and the PDA was unclosed after 6 weeks of life [2]. MCA included cerebral malformation, neural tube and spine defects, craniofacial and neck abnormalities, thorax abnormalities, ventral wall defect, abdomen and gastrointestinal tract defects, genitourinary malformation and skeletal dysplasia. ID/DD was diagnosed by the pediatric specialist. The peripheral blood from these children and their parents were collected. Informed consent was obtained from all the participants. The study was approved by the ethics committees of Guangzhou Women and Children’s Medical Center.

According to the National Birth Defects Prevention Study [20, 21], CHD was classified into the following types: septal defect, atrioventricular septal defect (AVSD), conotruncal defects, left ventricular outflow tract obstruction (LVOTO), right ventricular outflow tract obstruction (RVOTO), LVOTO + RVOTO, single ventricle (SV), and PDA (Table 1).

**Table 1:** Data of pathogenic copy number variants (PCNVs) in children with congenital heart disease (CHD) and CHD classification

CHD Classification	Simple CHD			Associate CHD			Total		
	No. PCNVs	No. tested	PR (%)	No. PCNVs	No. tested	PR (%)	No. PCNVs	No. tested	PR (%)
Septal defect	9	37	24.3	1	6	16.8	10	43	23.3
ASD	5	18	27.8	0	0	0	5	18	27.8
VSD	4	19	21.1	0	0	0	4	19	21.1
VSD + ASD	0	0	0	1	6	16.8	1	6	16.8
AVSD	0	1	0	0	2	0	0	3	0
Conotruncal defects	1	3	33.3	1	13	7.8	2	16	12.5
TOF	1	3	33.3	1	2	50	2	5	40
D-TGA	0	0	0	0	11	0	0	11	0
LVOTO	2	5	40	3	9	33.3	5	14	35.7
COA	0	2	0	1	4	25	1	6	16.7
AS	2	3	66.7	0	1	0	2	4	50
IAA, A	0	0	0	2	2	100	2	2	100
COA + AS	0	0	0	0	2	0	0	2	0
RVOTO	3	3	100	2	8	25	5	11	45.5
PS/PA	3	3	100	2	8	25	5	11	45.5
LVOTO + RVOTO	0	0	0	3	4	75	3	4	75
AS + PS	0	0	0	3	4	75	3	4	75
Single Ventricle	0	0	0	0	1	0	0	1	0
PDA	4	12	33.3	0	0	0	4	12	33.3
Total	19	62	30.6	10	43	23.2	29	104	27.9

*PR* positive rate, *ASD* atrial septal defect, *VSD* ventricular septal defect, *AVSD* atrioventricular septal defect, *TOF* tetralogy of Fallot, *D-TGA* d-transposition of the great arteries, *COA* coarctation of the aorta, *AS* aortic stenosis, *IAA* interruption arterial arch, *PS* pulmonary stenosis, *PA* pulmonary atresia, *PDA* patent ductus arteriosus, *LVOTO* Left ventricular outflow tract obstruction, *RVOTO* Right ventricular outflow tract obstruction

According to the complexity of CHD, CHD was divided into two groups: simple CHD and complex CHD [22]. Simple CHD are defined as anatomically discrete (e.g., ventricular septal defect, VSD) or a well-recognized single entity (e.g., tetralogy of Fallot, TOF). Complex CHD are defined as combinations of different heart defects (single ventricle was included in this group).

According to whether the children with CHD have ID/DD and/or MCA, CHD was divided into two types: isolated CHD and syndromic CHD. The syndromic CHD contained CHD plus MCA, CHD plus ID/DD, and CHD plus MCA and ID/DD.

### Chromosome microarray analysis

CMA was performed using Affymetrix CytoScan HD arrays according to manufacturer’s instructions. The procedure included genomic DNA extraction, digestion and ligation, PCR amplification, PCR product purification, quantification and fragmentation, labeling, array hybridization, washing and scanning. Data was analyzed with Chromosome Analysis Suite software version 1.2 (Affymetrix). The reporting threshold was set at 100 kb with marker count ≥50. CNVs were interpreted as benign listed in Database of Genomic Variants (DGV) database (*n* > 3 studies or documented in >1% of the normal population) or no gene included (not close to known CHD genes within 1 Mb) [23]. CNVs was classified as pathogenic copy number variations (PCNVs) if these CNVs overlapped completely with the minimal critical region of a well-known microdeletion or microduplication syndrome, or if the CNV comprised a dosage-sensitive gene known to cause a similar phenotype, referring to ClinGen Dosage Sensitivity Map (http://www.ncbi.nlm.nih.gov/projects/dbvar/clingen/). Rare CNVs (<1% in normal population and without OMIM genes) that do not meet the criteria above, should be considered as variant of uncertain clinical significance (VOUS), until proven otherwise (e.g. by functional studies) [8, 23]. For interpretation of these results, our in-house database and the following public database were used: DGV (http://projects.tcag.ca/variation/), Cytogenomics Array Group CNV Database (https://www.cagdb.org), Database of Chromosomal Imbalance and Phenotype in Humans using Ensembl Resources database (DECIPHER, http://decipher.sanger.ac.uk/), Online Mendelian Inheritance in Man (OMIM, http://www.omim.org), University of California Santa Cruz (UCSC, http://genome.ucsc.edu/, hg19), ClinVar (http://www.clinvar.com/) and CHD wiki (http://homes.esat.kuleuven.be/~bioiuser/chdwiki/index.php/Main_Page). All CNVs were further confirmed by Real-Time Polymerase Chain Reaction. Parental analysis were performed to interpret VOUS.

### Statistical analysis

Statistical analyses were performed using the Statistic Package for Social Science (SPSS) 13.0 statistical software package (SPSS Inc.). The PCNVs detection rates in children with simple CHD and complex CHD were compared using chi square test. Frequencies of recurrent CNVs in this study and the control cohorts were compared using fisher’s exact test. *P* value <0.05 was statistically significant. The PCNVs detection rates in isolated CHD, CHD plus MCA, CHD plus ID/DD, and CHD plus MCA and ID/DD were pairwise compared using chi square test or fisher’s exact test, *p* value <0.008 was statistically significant.

## Results

CMA was performed in 104 children (67 males and 37 females) with CHD aged from 5 days to 8 years old. CNVs were identified in 96.2% (100/104) of the children. The size of CNVs ranged from 102 kb to 13.8 Mb. CNVs were interpreted as benign or likely benign in 69.2% (72/104) children. The detection rate for PCNVs was 27.9% (29/104), and the VOUS rate was 2.9% (3/104) after parental analysis.

Detailed CHD classification and demographic data of the children were listed in Table 1. The percentages of children with simple CHD and complex CHD were 58.7% (61/104) and 41.3% (43/104), respectively. PCNVs were identified in 31.1% (19/61) children with simple CHD and in 23.2% (10/43) children with complex CHD. Pearson Chi-square test showed that there was no significant difference between these two groups (*P* > 0.05). PCNVs were detected in 23.3% (10/43) of septal defect, 0 (0/3) of AVSD, 12.5% (2/16) of conotruncal defects, 35.7% (5/14) of LVOTO, 45.5% (5/11) of RVOTO, 75% (3/4) of LVOTO + RVOTO, 0 (0/1) of SV, and 33.3% (4/12) of PDA.

The detection rates of PCNVs in isolated CHD and syndromic CHD were 17.9% (7/39) and 33.8% (22/65), respectively. There was no significant difference between these two groups (*P* > 0.05). The detection rates for PCNVs were 20% (5/25) in CHD plus MCA, 63.2% (12/19) in CHD plus ID/DD, and 23.8% (5/21) in CHD plus MCA and ID/DD, respectively (Table 2). The PCNVs rate of CHD plus ID/DD was significantly higher than that of isolated CHD (63.2 vs 17.9%, *P =* 0.001) and CHD plus MCA (63.2 vs 20%, *P* = 0.004).

**Table 2:** Classification of children with CHD and/or other diagnoses (MCA, ID/DD)

Classification of symptoms	Simple CHD			Complex CHD			Total		
	No. pCNVs	No. tested	PR (%)	No. pCNVs	No. tested	PR (%)	No. pCNVs	No. tested	PR (%)
Isolated CHD	3	15	20.0	4	24	16.7	7	39	17.9
Syndromic CHD	16	46	34.8	6	19	31.6	22	65	33.8
CHD + MCA	4	18	22.2	1	7	14.3	5	25	20
CHD+ ID/DD	8	13	61.5	4	6	66.7	12	19	63.2
CHD + MCA+ ID/DD	4	15	26.7	1	6	16.7	5	21	23.8
Total	19	61	31.1	10	43	23.2	29	104	27.9

*PR* positive rate, *MCA* multiple congenital anomalies, *ID/DD* intellectual disabilities/development delay

PCNVs were identified in 29 children (Table 3, including 22q11 deletion syndrome (*n* = 6), 22q11 duplication syndrome (*n* = 1), Williams-Beuren syndrome (*n* = 6), Angleman/Prader-Willi syndrome (*n* = 1), Wolf-Hirschhorn syndrome (*n* = 2), Schinzel-Giedion midface retraction syndrome (*n* = 1), 15q24 recurrent microdeletion syndrome (*n* = 1), 1p36 microdeletion syndrome (*n* = 1), Cornelia de Lange syndrome 4(*n* = 1), Marfan syndrome (*n* = 1), Opitz G/BBB syndrome (*n* = 1), 6q24 LOH (*n* = 1), and CNVs overlapped with DECIPHER patient entries (*n* = 6).

**Table 3:** Pathogenic copy number variants and variants of unknown significance detected by Chromosome Microarray Analysis in children with CHD

Child	Age^a^	Phenotype		CNVs: Region and size	Known syndrome/Decipher number/OMIM number	Significant genes (bold fonts) /candidate genes relating to CHD^b^
		Cardiac diagnosis	MCA or ID/DD			
Pathogenic CNVs						
1	8 y	ASD	ID	Dup 11q24.2-q25 (8.5 Mb)	Decipher number 255590	None
				Del 1q43-q44 (6.2 Mb)	Decipher number 284767	None
2	7 m	PS + ASD	None	Del 22q11.21 (3.2 Mb)	22q11 deletion syndrome	***TBX1***
3	10 m	PDA	Congenital anal atresia + DD	Dup 3p26.1-p24.3 (13.8 Mb)	Decipher number 260758	***CRELD1*** *;* ***RAF1***
				Del 6q13-q14.1 (5.2 Mb)	Decipher number 249539	None
				Dup 17q12 (1.4 Mb)	Decipher number 278456	None
4	4 y	PDA	Leukodystrophy	Del 1p36.33-p36.31(4.8 Mb)	1p36 microdeletion syndrome	*DVL1;SKI*
5	13 m	ASD	DD	Del 15q24.1-q24.2 (3.1 Mb)	15q24 recurrent microdeletion syndrome	***STRA6***
6	5 y	PS	ID	Del 15q11.2-q13.1 (4.9 Mb)	Angelman/Prader-Willi syndrome	None
7	5 m	VSD	DD	Del 4p16.3-p16.2 (5.7 Mb)	Wolf-Hirschhorn syndrome	***EVC2; EVC***
8	2 m	ASD	Laryngeal cartilage dysplasia	Del 22q11.21 (2.4 Mb)	22q11 deletion syndrome	***TBX1***
9	2 y	VSD	ID	Del 22q11.21 (3.2 Mb)	22q11 deletion syndrome	***TBX1***
10	14 m	ASD	None	Del 22q11.21 (1.4 Mb)	22q11 deletion syndrome	***TBX1***
11	2 y	ASD	Cleft palate + ID	Dup 18q12.3 (0.64 Mb)	Schinzel-Giedion midface retraction syndrome	*SETBP1*
12	2 y	AS + PS	DD	Del 7q11.23 (1.4 Mb)	Williams-Beuren syndrome	***ELN***
13	3 y	TOF + PLSVC + Pericardial defect	Diaphragmatic hernia + ID	Dup 2q12.3 (0.42 Mb)	Decipher number 287980	None
14	1 m	IAA,A + VSD	fingers of both hands and left toe deformity	Dup Xp22.2 (0.72 Mb)	Opitz G/BBB syndrome	***MID1***
15	1 y	PS + VSD	ID	Del 22q11.21 (3.2 Mb)	22q11 deletion syndrome	***TBX1***
16	15 m	PDA	DD	Del 1p36.33 (0.35 Mb)	Decipher number 106	None
				Dup 17q25.1-q25.3 (6.4 Mb)	Decipher number 249584	None
17	18 m	ASD	Cleft palate + DD	Del 4p16.3-p16.1 (7.6 Mb)	Wolf-Hirschhorn Syndrome	***EVC2; EVC***
18	11 d	IAA, A + VSD	None	Del 7q11.23 (1.4 Mb)	Williams-Beuren syndrome	***ELN***
19	8 m	AS + PS	DD	Del 7q11.23 (1.4 Mb)	Williams-Beuren syndrome	***ELN***
20	2 y	PS	None	Dup 15q21.1 (1.58 Mb)	Marfan syndrome	***FBN1***
21	3 y	TOF	Absence of corpus callosum + cerebellar vermis hypoplasia + ID	Del 1q43-q44 (7.6 Mb)	Decipher number 249647	None
				Dup 10p15.3-p14 (6.7 Mb)	Decipher number 278831	None
22	3 y	COA + VSD + ASD	ID	LOH 6q24.1-q24.2 (5.2 Mb)	Decipher number 290225	***CITED2***
23	1 m	AS	Hemivertebra + Adduction deformity of thumb + Polydactyly + Funnel chest	Del 8q23.3-q24.11(1.24 Mb)	Cornelia de Lange syndrome 4	*RAD21*
				Dup 11p15.3-15.2 (0.75 Mb)	None	None
24	2 y	VSD	ID	Del 22q11.21 (3.2 Mb)	22q11 deletion syndrome	***TBX1***
25	2 y	PS	ID	Del 7q11.23 (1.5 Mb)	Williams-Beuren syndrome	***ELN***
26	2 m	AS	None	Del 7q11.23 (1.5 Mb)	Williams-Beuren syndrome	***ELN***
27	2 m	AS + PS	None	Del 7q11.23 (1.4 Mb)	Williams-Beuren syndrome	***ELN***
28	7 m	PDA	Cleft palate	Dup 22q11.21 (2.5 Mb)	22q11 duplication syndrome	***TBX1***
29	6 m	ASD + VSD	None	Dup 17q25.1-q25.3 (8.5 Mb)	Decipher number 254723	None
				Del 20q13.33 (1.3 Mb)	Decipher number 2615	None
Variants of unknown significance						
30	1 m	CoA + Heart Enlargement	None	Dup 11p15.4 (0.18 Mb)	None	*STIM1*
31	1 m	AS + VSD	None	Dup 10q21.3 (0.4 Mb)	None	*CTNNA3*
32	1 m	D-TGA + VSD + ASD	None	Dup 6q22.31 (0.36 Mb)	None	*PLN*

*ASD* atrial septal defect, *PDA* patent ductus arterious, *PS* pulmonary stenosis, *VSD* ventricular septal defect, *TOF* tetralogy of Fallot, *PLSVC* persistent left superior vena cava, *AS* aortic stenosis, *IAA,A* interruption arterial arch, A type, *COA* coarctation of the aorta, *D-TGA* d-transposition of the great arteries, *DD* development delay, *ID* intellectual disabilities

^a^Age column: y, years; m, months; d, days

^b^Accordion to CHD wiki (http://homes.esat.kuleuven.be/~bioiuser/chdwiki/index.php/Main_Page)and OMIM database (http://www.omim.org)

In this study, PCNVs in 79.3% (23/29) of the children contained genes contributing to CHD (Table 3). The genes responsible for syndromic CHD included *TBX1* (22q11 deletion syndrome), *ELN* (Williams-Beuren syndrome), *EVC2* and *EVC* (Wolf-Hirschhorn syndrome), *STRA6* (15q24 recurrent microdeletion syndrome), *FBN1* (Marfan syndrome), *MID1* (Opitz G/BBB syndrome), *RAD21* (Cornelia de Lange syndrome 4) and *SETBP1* (Schinzel-Giedion midface retraction). In addition, the genes contributing to non-syndromic CHD included *CRELD1, RAF1* and *CITED2*. D*VL1* and *SKI* were identified as candidate genes for CHD in the current study.

CNVs detected in 9 children were classified as VOUS initially, further parental microarray analysis showed that CNVs in 6 children were inherited. The remaining CNVs in the other 3 children (Table 3, child 30-32) were de novo and the clinical significance was still unknown. Therefore, the VOUS rate was 2.8% in this study. The three VOUS were 11p15.4 duplication (chr11:3,923,985-4,242,111, 180 kb), 10q21.3 duplication (chr10:69,026,332-69,430,434, 400 kb) and 6q22.31 duplication (chr6:118,693,553-119,050,523, 360 kb). Three genes, *STIM1*, *CTNNA3* and *PLN* relevant with CHD, located in these fragments respectively. For the other children with benign or pathogenic CNVs, their parents rejected further parental analysis by CMA.

## Discussion

In the past few years, several studies have investigated postnatal cases with syndromic CHD by array CGH (aCGH) (Table 4) [9, 11, 13, 14, 16–19]. Different arrays were used in these studies, and the PCNVs detection rate ranged from 10.9 to 25.5%. Bachman’s study showed that the lowest PCNVs detection rate was 10.9% (5/46) using Roche NimbleGen 135 K arrays [18]. The highest detection rate was 25.5% (5/20) by Agilent 244 K array from Syrmou’s study [19]. In our study, the total detection rate for PCNVs reached 27.9% (29/104) by CytoScan HD array, including 1,950,000 oligonucleotide probes and 750,000 SNP probes. Our results demonstrated further that denser arrays with high resolution will lead to a proportional increase in number of PCNVs [13, 24].

**Table 4:** The comparison of studies in postnatal patients with isolate/syndromic CHD

Study	Array type	No. tested	Other diagnosis	PCNVs rates	VOUS rates
Thienpont et al. 2007 [9]	1 Mb BAC	60	MCA, ID	16.7%	11.7%
Richards et al. 2008 [11]	385 K oligo arrays	20	MCA, DD/ID	25%	9.8%
		20	None	0	
Erdogan et al. 2008 [10]	1 Mb BAC or 1 × 244 K Agilent arrays	105	None	4.7%	Unknow
Greenway et al. 2009 [12]	Affymetrix SNP 6.0	114	None	5.3%	Unknow
Breckpot J et al. 2010 [13]	1 Mb array	150	MCA	17.3%	Unknow
Rauch et al. 2010 [14]	100 K Affymetrix	19	MCA	21%	Unknow
Goldmuntz et al. 2011 [16]	100 K Oligo array	58	MCA	20.7%	3.4%
Breckpot et al. 2011 [15]	Affymetrix SNP 6.0	46	None	4.3%	Unknow
Derwinska et al. 2012 [17]	180 K Oligo	150	MCA	14%	Unknow
Syrmou et al. 2013 [19]	1 × 244 K or 4 × 180 K Agilent arrays	55	MCA	25.5%	Unknow
Bachman et al. 2013 [18]	Roche NimbleGen 135 K arrays	46	MCA	10.9%	Unknow
Our study	Affymetrix CytoScan HD arrays	104	MCA, ID/DD	27.9%	2.9%

*BAC* bacterial artificial chromosome, *MCA* multiple congenital anomalies, *DD/ID* development delay/intellectual disabilities, *SNP* single nucleotide polymorphism

CMA has also been applied in children with isolated CHD previously [10–12, 15]. The PCNVs detection rates ranged from 0 to 5.3% (Table 4). Richards et al. studied 20 children with isolated CHD and 20 children with CHD plus MCA, the results showed that PCNVs detection rate in isolated CHD was 0 and that in CHD plus MCA was 25%, and the highest PCNVs detection rate was 45% in children with CHD plus neurologic defects [11]. Therefore, CMA was not recommended by Richards for children with isolated CHD. In our study, PCNVs detection rate was 17.9% (7/39) in isolated CHD. The PCNVs detection rates for CHD plus MCA, CHD plus ID/DD and CHD plus MCA and ID/DD were 20, 63.2 and 23.8%, respectively. PCNVs detection rate in CHD plus ID/DD was significantly higher than that of isolated CHD (*P* = 0.001) and CHD plus MCA (*P* = 0.004). Our data demonstrated that CMA is the most useful for genetic diagnosis in children with CHD plus ID/DD. In addition, we also recommended CMA investigation for children with isolated CHD.

In this study, PCNVs were detected in 31.1% (19/61) children with simple CHD and 23.2% (10/43) children with complex CHD by CMA. There was no significant difference between the two groups (*P* > 0.05). Detection rates in various types of CHD were different. Shaffer et al. reviewed 580 fetuses with CHD and normal karyotype by aCGH [25], and revealed the detection rates of PCNVs as follows: 16.2% (11/68) in LVOTO, 11.6% (5/43) in conotruncal defect and 10.6% (14/132) in septal defect. The above three types of CHD in fetuses were the most frequent. In our study, the PCNVs detection rates in different types of CHD in isolated or with additional anomalies were as follows in turn: 75% (3/4) in LVOTO + RVOTO, 45.5% (5/11) in RVOTO, 35.7% (5/14) in LVOTO, 33.3% (4/12) in PDA, 23.3% (10/43) in septal defect, 12.5% (2/16) in conotruncal defects. Our data demonstrate that LVOTO and/or RVOTO were most probably related to microdeletion/microduplication. Of the 29 children with PCNVs, 22 (75.9%) were complicated with MCA and/or DD/ID. High detection rate in children with PDA (33.3%) was obtained in this study, and we noticed that all the 12 children with PDA were complicated with MCA and/or ID/DD. There was no PCNVs detected in children with AVSD (*n* = 2) and SV (*n* = 1), this may be due to the small sample size in our study.

Gijsbers et al. suggested that as the rising of aCGH resolution, there will be more VOUS identified [26]. In the previous studies in children with CHD by microarray, the VOUS detection rates ranged from 3.4 to 11.7% [9, 11, 16]. In this study, CNVs detected in 9 children were unknown of clinical significance. After parental microarray analysis, CNVs in 6 children were inherited, the remaining CNVs in the other 3 children (Table 3, child 30-32) were de novo and the clinical significance was still unknown. Finally, the VOUS rate was 2.9% in our study, which did not increase obviously as the resolution rise compared with previous studies. Therefore, parental analysis could assist in interpreting CNVs and reducing VOUS rate.

The clinical features of 1p36 microdeletion syndrome include microcephaly, brachycephaly, developmental delay with hypotonia, seizures and cardiac defects [27]. In our study, CMA revealed a 4.8 Mb deletion at 1p36.33-p36.31 (chr1:849,466-5,685,789) in child 4 with PDA and leukodystrophy, which contained the *SKI* and *DVL1* genes. *SKI* morphant embryos showed severe cardiac anomalies, especial complete failure in cardiac looping and malformations of the outflow tract [28]. Researchers have studied *DVL1* null mice and reported that *DVL1*-mediated planar cell polarity signal was crucially for cardiac outflow tract development [29]. Based on the above data, *SKI* and *DVL1* could be the main genes responsible for CHD phenotypes in 1p36 deletion syndrome.

A 4.9 Mb deletion at 15q11.2-q13.1 (chr15: 23,620,191- 28,540,345) was detected in child 6 (Table 3) with pulmonary stenosis plus ID. He was diagnosed as Angelman/Prader-Willi syndrome, the clinical features include ID, microcephaly, seizures, truncal ataxia, feeding difficulties in infancy and muscular hypotonia. Soemedi et al. first reported the association of 15q11.2 deletion with CHD, in their study the phenotypes contained left-side malformations, coarctation of the aorta, septal defect and TOF [30]. Geng et al. have detected four patients with 15q11.2 deletion (size range 245-2703 kb) in 502 CHD patients, their result also showed the 15q11.2 deletion has low penetrance in CHD patients [31]. These data indicated that 15q11.2 deletion, containing the Angelman/Prader-Willi syndrome region, represents a critical CHD locus. However, further studies are need to ascertain the genes responsible for CHD.

In the present study, we identified two children with 1q43-q44 deletion. Child 1 manifested atrial septal defect (ASD) and ID. Child 21 manifested TOF and absence of corpus callosum. Our study showed significantly higher frequency of 1q43-q44 deletion (2/104) than that of the 9170 control cases (4/9170) (*P* = 0.002) [30, 32]. Therefore, 1q43-q44 should be considered as a locus associated with cardiac development. The clinical features of 1q43-44 deletion syndrome include ID, language retardation, characteristic facial features, abnormalities of the corpus callosum and seizures [33]. Summarizing the literature, patients with 1q43-q44 deletion manifesting ID/DD or structural anomaly of central nervous system (69/83, 83.1%) more frequently than CHD (19/76, 25%) (*P* < 0.001, Table 5). These data suggested 1q43-q44 deletion may have low penetrance in CHD children.

**Table 5:** 1q43-q44 deletion in our study compared with other studies

Phenotype	Our case	1q43 deletion^a^	1q44 deletion^b^	1q43-q44 deletion^c^	1q42-q44 deletion^d^	Total
ID/DD or CNS abnormal	2/2	22/27	24/24	15/24	6/6	69/83
Congenital heart defects	2/2	8/24	5/22	2/24	2/4	19/76

*ID/DD* intellectual disabilities/development delay, *CNS* central nervous system

^a^Reference to [42–45]

^b^Reference to [42–44, 46–48]

^c^Reference to [33, 49]

^d^Reference to [44]

The child 11, a girl with ASD and cleft palate which had been corrected surgically. She was diagnosed with mental retardation at 2 years old by pediatric doctor. CMA test revealed chromosome 18q12.3 duplication (chr18: 41,814,626- 42,453,303). *SETBP1* gene located in the fragment which was responsible for Schinzel-Giedion midface retraction syndrome, and point mutation is the most common type. The clinical features included severe mental retardation, distinctive facial features, and multiple congenital malformations such as skeletal abnormalities and cardiac defects. A dominant-negative or gain-of-function mechanism was proposed to underlie this syndrome [34]. We sequenced the 5 exons of *SETBP1* gene, and no mutation was detected. The *SETBP1* gene duplication was in accordance with gain-of-function mechanism, and the child’s manifestations were similar with the phenotypes of Schinzel-Giedion midface retraction syndrome, Therefore, *SETBP1* should be the main responsible gene for this patient.

The child 22 had coarctation of aorta, VSD, ASD (cardiac surgery was performed when he was 1 month old) and ID. Brain MRI showed normal result. CMA test revealed a 5.2 Mb LOH on chromosome 6q24.1-q24.2 (chr6:139,184,381-144,411,648). This LOH fragment included *PLAG1* and *HYMAI* genes, which were associated with imprinting disorder intrauterine growth retardation and neonatal hyperglycemia [35]. Another gene *CITED2* also located in the LOH, which has been identified associated with cardiac malformations, including atrial and ventricular septal defects, overriding aorta, double-outlet right ventricle and right-side aortic arches [36]. Therefore, this fragment was classified as PCNVs.

Of the 29 children with PCNVs, 7 (24.1%) were with 22q11 deletion (*n* = 6) or duplication (*n* = 1) syndrome, which was the most common type. The incidence of 22q11 deletion/duplication was 6.7% (7/104) in the children with CHD in the present study. These data indicate that in addition to CMA it could be more cost-effective to exclude 22q11 deletion/duplication firstly by targeted technique such as MLPA in children with CHD.

In the 3 children with VOUS, we identified CHD candidate genes such as *STIM1, CTNNA3* and *PLN*. *STIM1* gene mutation could cause cardiomyocyte hypertrophy [37]. *PLN* gene mutation was associated with cardiomyopathy [38]. *CTNNA3* gene mutation could cause arrhythmogenic right ventricular dysplasia, compound heterozygous deletion was related to ASD [39, 40]. However, the children’s symptoms in this study were inconsistency with genotypes in the database. Further study of these genes are still needed to evaluate the clinical implication.

## Conclusion

CMA has been recommended as a first-tier clinical diagnosis test in patients with DD, ID and/or MCA [8, 41]. Study from Bachman also recommended aCGH as a first-tier test in evaluation of neonates with CHD [18]. In the present study, we obtained the highest detection rate in children with CHD plus ID/DD (63.2%), while in children with isolated CHD we still obtained relatively high detection rate (17.9%). Based on the above data, we suggest CMA as a first-tier test in children with CHD, especially in children with CHD plus ID/DD.

## Abbreviations

aCGH: Array CGH
AOH: Absence of heterozygosity
ASD: Atrial septal defect
AVSD: Atrioventricular septal defect
CHD: Congenital heart disease
*CITED2*: Cbp/p300-interacting transactivator, with Glu/Asp-rich carboxy-terminal domain, 2
CMA: Chromosome microarray analysis
CNVs: Copy number variations
*CRELD1*: Cysteine-rich with EGF-like domains 1
*CTNNA3*: Catenin (cadherin-associated protein), alpha 3
*CYFIP1*: Cytoplasmic FMR1 interacting protein 1
DD: Developmental delay
DECIPHER: Database of chromosomal imbalance and phenotype in humans using ensembl resources
DGV: Database of genomic variants
*DVL1*: Dishevelled segment polarity protein 1
*ELN*: Elastin
*EVC*: Ellis van Creveld syndrome
*EVC2*: Ellis van Creveld syndrome 2
*FBN1*: Fibrillin 1
*HYMAI*: Hydatidiform mole associated and imprinted (non-protein coding)
ID: Intellectual disabilities
LVOTO: Left ventricular outflow tract obstruction
MCA: Multiple congenital anomalies
*MID1*: Midline 1
*NIPA1*: Non imprinted in Prader-Willi/Angelman syndrome 1
*NIPA2*: Mon imprinted in Prader-Willi/Angelman syndrome 2
OMIM: Online mendelian inheritance in man
PCNVs: Pathogenic copy number variations
PDA: Patent ductus arteriosus
*PLAG1*: Pleiomorphic adenoma gene 1
*PLN*: Phospholamban
*RAD21*: RAD21 homolog
*RAF1*: Raf-1 proto-oncogene, serine/threonine kinase
RVOTO: Right ventricular outflow tract obstruction
*SETBP1*: SET binding protein 1
*SKI*: SKI proto-oncogene
*STIM1*: Stromal interaction molecule 1
*STRA6*: Stimulated by retinoic acid 6
SV: Single ventricle
*TBX1*: T-box 1
*TTN*: Titin
*TUBGCP5*: Tubulin, gamma complex associated protein 5
UCSC: University of California Santa Cruz
VOUS: Variant of uncertain clinical significance
VSD: Ventricular septal defect
